# Treating chronic diarrhea: A systematic review on Immunoproliferative Small Intestinal Disease (IPSID)

**DOI:** 10.1371/journal.pone.0253695

**Published:** 2021-07-16

**Authors:** Daniele Evangelista-Leite, Breno Affonso Madaloso, Bruno Shouta Yamashita, Francesco Enrico Aloise, Lucas Polito Verdasca, Murilo Lopes de Mello, Renan Murata Hayashi, Ethel Zimberg Chehter

**Affiliations:** Department of Gastroenterology, School of Medicine, Faculdade de Medicina do ABC, Santo André, São Paulo, Brazil; University of Illinois at Chicago, UNITED STATES

## Abstract

Immunoproliferative Small Intestinal Disease (IPSID) is a disease characterized by extra-nodal marginal zone B-cell lymphoma with villous atrophy in the small intestine, causing chronic intermittent non-bloody diarrhea. Although originally associated with the Mediterranean region, this disease is present in many countries worldwide and may have been underreported due to its complicated diagnosis and scarce scientific literature, especially in regards to treatment. This study aims to review IPSID clinical features, therapeutic options, and treatment outcomes to help physicians identify and treat IPSID. Using PRISMA guidelines, a systematic review of articles was conducted on PubMed database with search terms including IPSID, therapy, treatment, and outcomes. Inclusion and exclusion criteria were used to select 33 English language articles published from the year 2000–2020 that included relevant clinical information about IPSID treatment. Data were extracted independently by at least two authors to reduce the introduction of potential bias. There were 22 case reports, 7 reviews, 1 research article, 1 prospective study, 1 letter to the editor and 1 memoriam in which 76 patients were identified. Epidemiological analysis showed a mean patient age of 32 years old, 2.4:1 mal to female ratio and heterogeneous ethnicities, with 16 Europeans (43.2%) and 12 Asians (32.4%). Chief symptoms included chronic diarrhea (53/76, 69.7%), weight loss (49/76, 64.4%), malabsorption (38/76, 50%), abdominal pain (32/76, 42.1%), and finger clubbing (24/76, 31.6%). Patients stratified into the early disease stage (Galian A) were treated with tetracycline antibiotics, corticosteroids, and non-pharmacological supplements with mostly with complete or partial remission. Late stages (Galian B or C), were treated mostly with anthracycline-based chemotherapy, and occasionally surgery, radiotherapy, or rituximab. This work offers a targeted approach to diagnosing and treating IPSID to aid physicians and serve as a treatment guideline recommendation for future public policies and clinical studies.

## Introduction

Immunoproliferative Small Intestinal Disease (IPSID) is a rare extra-nodal marginal zone B-cell lymphoma, a variant of mucosa-associated lymphoid-tissue lymphoma (MALT Lymphoma). IPSID has had many synonyms due to its broad symptomatic spectrum. In the early 1960s, young adults from Israel were reported to present small intestinal lymphoma associated with malabsorption symptoms, coining the term Mediterranean Lymphoma to this disease, as it was predominantly found in patients from that geographical region [1, 2]. In 1965, Bracha was the first to describe 13 patients in Tel-Hashomer hospital with this syndrome [3, 4]. In 1968, Mediterranean lymphoma was associated with the term α-heavy chain disease due to discovering an abnormal IgA molecule in patients’ serum and other body fluids [1]. This IgA molecule lacked light chains and had a truncated alpha-heavy chain protein, which rendered this disease a new name: “alpha-chain disease” [1, 5, 6]. Alpha chain disease may affect different anatomical structures, dividing it into three types: digestive, respiratory, and lymphoid [5]. The digestive alpha chain disease is now called Immunoproliferative Small Intestinal Disease.

Although initially associated with the Mediterranean, IPSID is found worldwide due to poverty and poor sanitation [6, 7], and improvements in living conditions reduced its incidence [8]. IPSID has only a few cases reported globally [9], but has a higher incidence in children and young adults in the second and thirds decades of life [2, 5, 6]. There is no clear preference for a specific ethnicity or race, but the incidence was higher in men with a male to female ratio of 2.4:1 [2]. Its clinical features include chronic intermittent non-bloody diarrhea associated with nutrient malabsorption, abdominal pain, weight loss, and finger clubbing [10, 11]. Because these are common symptoms of other diseases, diagnosis is challenging. Although IPSID is hardly the first diagnostic hypothesis, doctors should be aware of this possibility in their differentials to search for pathological features such as CD20 positive lymphoid cells and CD138 positive plasma cells in intestinal biopsies that allow for the correct diagnosis [2].

The number of publications on this topic is scarce and generally describes isolated or regional cases. Systematically revising the information from the last 20 years regarding IPSID would be beneficial for building up-to-date clinical guidelines and guiding therapeutics. Therefore, this work revises IPSID clinical features, therapeutic options, and treatment outcomes from the few articles that have been published in the last 20 years in order to analyze treatments with the best outcomes. The final goal is to produce a treatment flowchart divided by disease stages, which may aid health professionals, students, and public policy advocates when considering IPSID patients.

## Methodology

This review aims to answer the following question: *What are the most used treatment options with the most favorable outcomes reported globally in the last 20 years for treating IPSID patients*?

Although no review protocol was registered, a systematic search following PRISMA 2009 guidelines [12] was conducted for potentially relevant studies discussing different IPSID treatment regimens. The stepwise approach for this review was: identifying the goal, choosing keywords, establishing eligibility criteria, searching for articles in the database, and screening articles by first adjusting at the advanced search parameters on the database, reading the abstract, applying exclusion criteria, and finally reading the entire article before deciding to include it in the review. Inclusion criteria were any type of literature like academic articles, case reports, reviews, short communication and letters to the editor. Articles published before January 1^st^, 2000, not written in English, unavailable for download, and lacking clinical information about IPSID treatment were excluded. Article search was conducted in Pubmed starting on September 4^th^, 2020 and ending on September 6^th^, 2020. The following search terms were used: “((IPSID) or (immunoproliferative small intestine disease)) and ((TREATMENT) or (OUTCOMES) or (TREAT) or (THERAPY)).” Database searches were supplemented with a search for unpublished (grey) literature, including a review of clinical trials via ClinicalTrials.gov and greylit.org, but no results were found on IPSID.

After applying inclusion and exclusion criteria, selected downloaded articles were stored in the cloud using Google Drive, and references were saved in the Endnote citation manager. No data abstraction tool was used. All authors independently searched articles and screened abstracts. Full article screening to select the final articles was decided by all seven reviewers together. To ensure reliable data detection and collection, more than one person extracted data from every article selected to minimize errors and reduce the introduction of potential biases by authors. Data extraction from reports was done by filling up tables electronically using Google drive. At the very least, information that involved IPSID treatment and outcomes was extracted independently by two people.

Due to the limited pool of articles, being most of them case reports, it was not possible to analyze the level of evidence, grade rating and bias analysis required in the PRISMA protocol Therefore, some items of the PRISMA checklist could not be applied here. All articles selected according to our search criteria were included in this review, despite their PRISMA rating grade, evidence level or bias. It was equally challenging to present their risk of bias, outcome level assessment and strength of evidence. Therefore, this review may lack some of this information, but the cases chosen were analyzed in detail.

## Results

### Study selection

**Fig 1:**
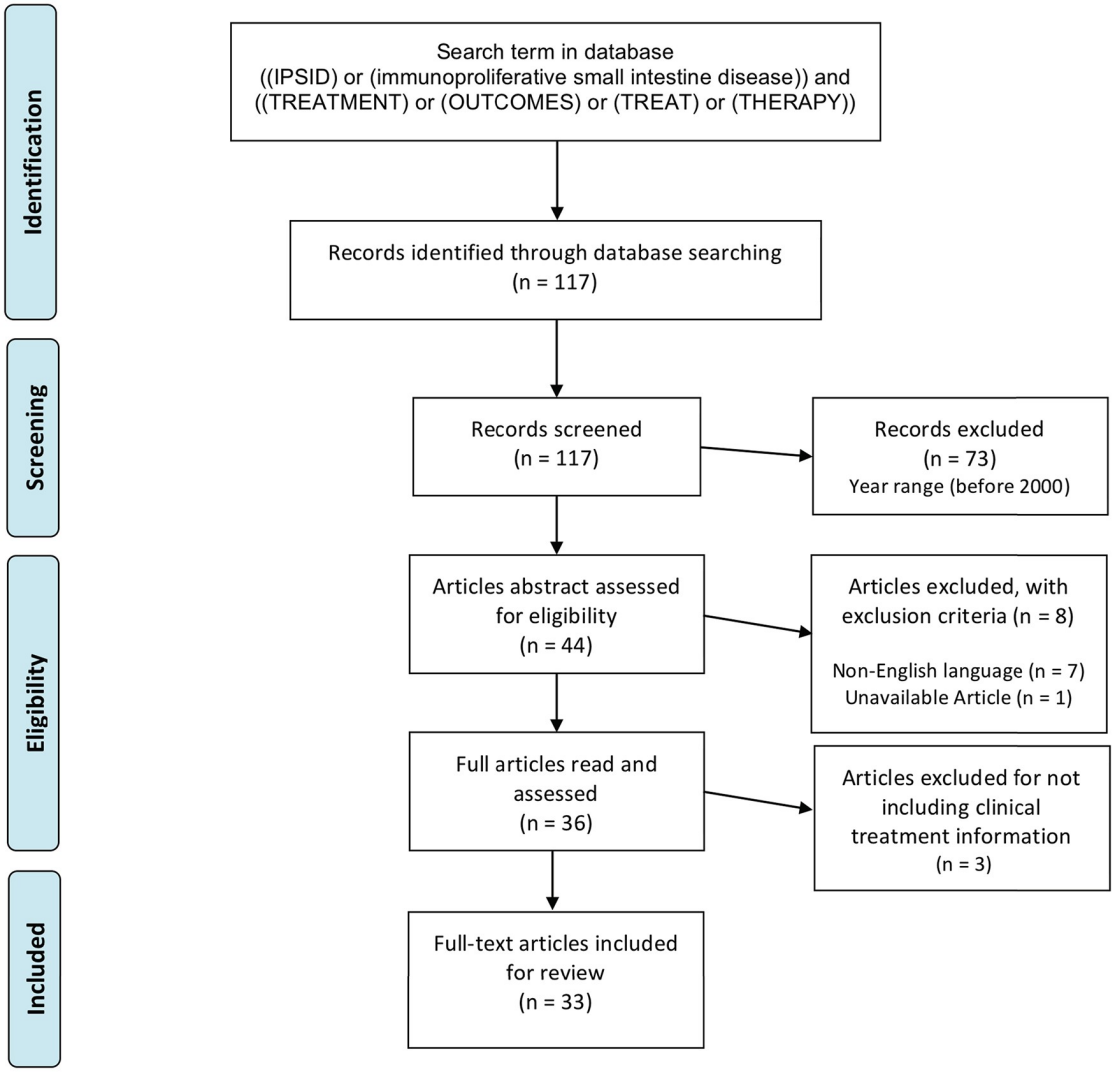
Inclusion and exclusion criteria following PRISMA protocol.

The Pubmed search first yielded 117 articles. Articles were screened and those published before January 1^st^, 2000 were excluded, resulting in 44 articles. Articles abstracts were revised to apply inclusion and exclusion criteria. In this step, five French articles, one German and one Chinese were excluded because of language and another for being unavailable to download. In total, 36 articles were eligible to read in full text and screen for relevant clinical information. Three articles were excluded because they did not discuss IPSID treatment, yielding 33 articles in this review (Fig 1).

### Study characteristics

In the 33 articles selected for the study, there were 22 case reports, 7 reviews, 1 research article, 1 prospective study, 1 letter to the editor and 1 memoriam. The case reports evaluated a total of 76 IPSID patients (Table 1). After analyzing patients’ age, sex and location, an epidemiological profile was drawn. IPSID patients had a mean age of 32 years old and were primarily men, in a male to female ratio of 2.4:1 (52 male, 20 female and 4 non-reported). A total of 65 patients reported their ethnicities, from which 28 were Middle Eastern (43%), 19 European (29%), 11 Asian (17%), 3 Oceanian (5%), 2 American (3%) and 2 African (2%).

**Table 1:** Features reported in IPSID cases.

Age	Sex	Origin	Clinical features	Exams	Treatment	Outcomes	Ref
Mean 28.7 ± 10.2	19M, 8F	Pakistan	Abdominal pain and/or diarrhea/ malabsorption (n = 11). Weight loss, vomiting, nausea, and abdominal mass (n = 16).	Elevated serum IgA. Endoscopy: polypoidal or nodular lesions, hyperemic raised or flat edematous lesions. Biopsy: blunting and shortening of villous architecture with flattening of mucosal folds, and diffuse lamina propria CD20+ lymphocyte and CD138+IgA+ plasma cell infiltration.	Tetracycline for Galian stages A and B. Six to eight cycles of CHOP chemotherapy for Galian stage C.	Partial or complete regression in early stages, with only 3 cases of transformation into diffuse large B-cell lymphoma.	[2]
35	F	China	Chronic watery diarrhea and weight loss for 3 years. Weight loss, rickets, anemia, and anxiety.	High serum IgA, severe electrolyte disorder, bone marrow aspiration showing sideropenic and megaloblastic anemia but no lymphoma. Serum protein electrophoresis with no monoclonal immunoglobulin protein band. Duodenum biopsy: atrophy, villi blunting and lymphoplasmocytic infiltration of the lamina propria. CT: diffuse thickening of small intestine wall.	Supplementation of vitamin D, calcium, magnesium, potassium, iron, folic acid, mecobalamin, and microflora probiotics for 1 month. Prednisone (40 mg/day) for 1 month.	During one month the probiotics improved the diarrhea, but did not resolve all symptoms. Prednisone resolved symptoms and restored weight loss.	[5]
79	M	Italy	Abdominal pain for 4 months, colic timpanists, tenderness, distention, weight loss, and diarrhea.	Hypochromic anemia. USG: steatosis and gallbladder lithiases. CT: esophageal wall thickening, expansive caecum mass and nodal mass near ileo-colic artery. Colonoscopy and biopsy: diverticulosis, villous adenoma, CD20+ lymphoplasmocytic infiltration. Positive for *H*. *pylori* and follicular B-cell lymphoma. Post-surgical biopsy: ileal T-cell lymphoma, chronic inflammatory disease in the ileum and colon cancer.	*H*. *pylori* eradication therapy; Right hemicolectomy, lymphadenectomy and cholecystectomy. No chemotherapy due to advanced age.	Good condition and remission after 5-year follow-up.	[13]
26	M	Marshall Islands	Chronic watery diarrhea (10–20 watery stool per day) for 6 months that had failed to respond to oral antibiotics, 40-lb weight loss weakness, and cachexia.	Leukocytosis, mild anemia, hypokalemia, hyponatremia, non-anion gap metabolic acidosis and multiple bacterial gastrointestinal infections, including *Campylobacter jejuni* and *Vibrio fluvialis* by stool culture. CT: mesenteric and retroperitoneal lymphadenopathy, hepatomegaly and intestinal edema. PET-CT: extensive small bowel and mesenteric hypermetabolic activity. Upper endoscopy: atrophic nodular mucosa in the duodenum. Colonoscopy: nodular mucosa in terminal ileum with whipworm in the cecum. Duodenal and terminal ileum biopsies: monotypic α-heavy chain lymphocytes expansion in the lamina propria, villi blunting and focal crypt destructive lesions. Gastric biopsy: *Helicobacter pylori*. Mesenteric lymph nodes with *Escherichia coli*.	Piperacillin-tazobactam for 14 days to treat *C*. *jejuni* and *V*. *fluvialis*, followed by 28 days of ceftriaxone with metronidazole, azithromycin, pantoprazole for 14 days to treat *E*. *coli* and *H*. *pylori*. Albendazole for whipworm. Moxifloxacin was used to keep the enteric pathogens suppressed.	Antibiotic treatment improved diarrhea and weight loss symptoms and controlled the bacterial infection, but did not stop lymphadenopathy progression. Rituximab was not sufficient to control IPSID spread to other regions such as the tonsils, transforming into diffuse large B-cell lymphoma.	[7]
					Four cycles of rituximab were used to treat the IPSID. Six cycles of R2-CHOP were used to treat diffuse large B-cell lymphoma.		
20–62, Median 38	8M, 5F	Greece (n = 11); Albania (n = 2)	Chronic diarrhea (n = 12), weight loss (n = 12), malabsorption (n = 10), finger clubbing (n = 7), intestinal perforation (n = 3), abdominal pain, intestinal pseudo-obstruction (n = 1), neurological manifestations (n = 1), cachexia (n = 1), and amenorrhea (n = 1).	Serum immunoglobulin positive for IgA and varying levels of IgM and IgG, α-heavy chain protein in blood. Alterations in small intestine biopsies	Galian stage A: Tetracycline and/or metronidazole, or antibiotics with CHOP. Galian stage B or C: antibiotics with cyclophosphamide and/or chemotherapy.	Three patients died of jejunum perforation. Ten treated with antibiotics and cytotoxic drugs, did not respond to treatment or died after relapse. Two are alive and being monitored. One was lost to follow-up.	[1]
16	M	Iran	Chronic diarrhea and crampy abdominal pain for 5 years, edema, ascites, malnutrition, and lymphadenopathy.	Leukocytosis, but other parameters were normal Ascites fluid contained 75% lymphocytes and was negative for pathogens. Duodenum mucosa biopsies: CD20+ lymphocyte infiltration with mild to moderate villi atrophy without changes to the epithelium.	Nutritional support, vitamins and mineral supplementation. Azithromycin and metronidazole for 6 months.	Symptom improvement in the first month and intestinal healing after 6 months of antibiotic treatment.	[14]
12	M	Turkey	Diarrhea, abdominal pain, and weight loss for 5 months, finger clubbing, edema, malnutrition, and dehydration.	Anemia due to iron deficiency, hypoalbuminemia, hypokalemia, low serum calcium, steatorrhea, impaired D-xylose absorption, and elevated IgA. Barium x-ray: segmentation, bowel dilation, and mucosal fold thickening. Abdominal ultrasonography and CT: enlarged mesenteric lymph nodes. Small intestine biopsy: vilous atrophy with lymphoplasmocytic infiltration of the lamina propria, enlarged follicles with poorly defined edges and plasma cells lacking kappa chain by immnunoperoxidase studies.	Tetracycline and gluten free diet. After recurrence, used cytotoxic regimen.	Tetracycline improved symptoms substantially and reduced the size of mesenteric lymph nodes. One year after patient stopped treatment, symptoms recurred. Disease evolved to malignant anaplastic cell infiltration resulting in death 2 weeks later, despite introduction of cytotoxic drugs.	[15]
**2**	M	Romania	Diarrhea, vomiting and anorexia for 2 months. Intermittent fever, enlarged liver, anemia and recurrent infections. Abdomen was swollen, painful, large and contained collateral circulation abdomen.	Lymphocytosis, hiposideremic anemia, elevated erythrocyte sedimentation rate, and positive stool occult bleeding test. Ultrasonography: enlarged liver, gallbladder and left kidney. X-ray: diffuse hand and forearm osteoporosis, hydroaeric levels in the superior and inferior quadrants. Jejunal biopsy: vilosity flattening, lympho-plasmocytic infiltration and chronic enteritis. Laparotomy: mesenteric adenopathies and thick intestinal wall. Lymph nodes were positive for *Staphylococcus albus* and enterococcus, and contained lymphoplasmocytic infiltration.	Laparotomy (initial clinical suspicion was Hirschprung disease)	Postoperative fever, tachycardia, dispneia, leading to death by cardio-respiratory arrest. IPSID diagnosis was done post-mortem.	[16]
**57**	M	Spain	Iron deficiency anemia for 6 months	Bone marrow analysis. Upper endoscopy: mucosal edema, friability and erythema. Intestinal biopsy: lympho-plasmocytic mucosal infiltration. Gastric biopsy: *H*. *pylori*. CT and capsule endoscopy: proximal small bowel involvement.	Tetracycline for 6 months following *H*. *pylori* eradication.	-	[17]
**35**	M	Mexican heritage, but born in the United States	Mild, intermittent, non-bloody diarrhea, steatorrhea, lipid intolerance, malabsorption, and frequent episodes of small bowel obstruction.	Elevated alkaline phosphatase, negative stool studies, and steatorrhea. Barium contrast X-ray: small intestinal dilation. Small intestine biopsy: plasma cell lamina propria infiltration, mucosal lymphocyte hyperplasia, villous widening, but no changes to the epithelium. Mucosa with predominantly IgA expressing B-cells accumulation. Exploratory laparotomy: lymphoid hyperplasia in mesenteric nodes.	Early non-lymphoma stage, treated initially with tetracycline, which was changed to a long term (30 years) corticosteroid regimen of prednisone at 10 mg/day.	Tetracycline reduced the diarrhea, but it was changed to prednisone for sustained clinical improvement. Although no lymphoma developed, there were several episodes of small bowel obstruction with villous blunting and epithelium flattening, leading to death by total obstruction 30 years after initial diagnosis.	[10]
**20**	M	India	Fever, anorexia, abdominal pain and weight loss (16kg) during the last 4 months. No history of diarrhea, colicky abdominal pain with postprandial exacerbation.	Elevated erythrocyte sedimentation rate, bacterial overgrowth in hydrogen breath test. Barium meal: nodularity and thickening of duodenal and jejunal folds. Contrasted CT: small intestinal thickening with mesenteric lymphadenopathy. Upper gastrointestinal endoscopy and enteroscopy: nodularity and thickening of folds with ulcerations. Gastric urease test: positive for *H*. *pylori*. Duodenum and jejunum biopsy: diffuse lymphoplasmacytic infiltration of the lamina propria (stage A). Laparotomy: enlarged mesenteric lymph nodes.	Doxycycline after *H*. *pylori* eradication therapy with amoxicillin (500 mg), clarithromycin (500 mg), lansoprazole (30 mg) for 7 days and amoxicillin (1 g), tinidazole, omeprazole (20 mg) and bismuth subcitrate for 14 days.	Responded well to *H*. *pylori* eradication therapy and had clinical improvement after 6 months, remaining symptom-free at 7 years of follow-up.	[18]
**45**	F	Cameroon	Chronic diarrhea and wasting for 12 months.	Lymphocytosis with 82% of peripheral-blood lymphocytes being CD19+, CD20-, CD38+, CD138- B cells with high levels of cytoplasmic α-heavy chain and no detectable light chain. Bone marrow biopsy with lymphoplasmacytes infiltration. Increased serum IgA and decreased IgM and IgG levels. Aspirated content from jejunum lumen contained α-heavy chain precipitin. Duodenum and jejunum biopsies: plasma cells and CD20+ centroyte-like lymphocytes infiltration of the lamina propria, mostly around crypts. Plasma cells and lymphocytes expressed only immunoglobulin α-heavy chain. Biopsies were positive for *Campylobacter jejuni*.	Antimicrobial therapy with amoxicillin (1 g, 2x/day), metronidazole (500 mg, 2x/day), clarithromycin (500 mg, 2x/day) and omeprazole (20 mg, 2x/day) for 5 months.	Diarrhea resolved after one week and lymphocytosis was normalized after 10 weeks. Full remission occurred after 5 months with normalized serum and biopsies specimens.	[19]
**52**	M	Japan	Abdominal pain, ileocecum obstruction, and fever.	Hypoalbuminemia and hypogammaglobulinemia. Abdominal X-ray: organomegaly, lymphadenopathy. Biopsy: massive infiltration of plasma cells and centrocyte-like lymphocytes in the lamina propria, with the former being more superficial and the latter deeper into the mucosa. There were CD45RO+CD5+ (T cells) and CD20+M1B-1+ (atypical B cells) lymphocytes. Upper gastrointestinal endoscopy: duodenal mucosal nodularity. Colonoscopy: broad terminal ileum erosion. Barium x-ray: disappearance of Kerckring folds, dilation and partial ileum stenosis. Abdominal CT: splenomegaly, enlarged mesenteric lymph nodes and thickening of ileum wall. Scintigraphy: hot spot on the left lower abdomen and colon protein leakage.	Surgical removal of ileocecum obstruction. Six courses of THP-COP therapy after initial diagnosis. Two cycles of R-P-IMVP16/CBDCA (rituximab, methylprednisolone, ifosfamide, methotrexate, etoposide, and carboplatin) after relapse. Salvage therapy with R-CdA (rituximab and cladribine).	Complete remission for 16 months after THP-COP. Later relapsed and evolved to unspecified peripheral T-cell lymphoma 2 years after onset. Patient condition degenerated, leading to sepsis and death.	[20]
**15–35, Median 27**	1M, 1F, 4NR	India	Chronic watery diarrhea, decreased appetite, fever and weight loss (median duration 13,5 months, range 6–26 months), abdominal pain, and clubbing.	Barium meal: ileo-ileal intussusception (n = 2), CT: oedematous mucosal thickening in small intestine and enlarged mesenteric lymph nodes (n = 6). Biopsy: diffuse CD20+CD3- lymphoplasmacytic mucosal infiltration with partial or total villous atrophy.	Galian stage A: CHOP, CHOP with tetracycline, antibiotics with doxycycline, antibiotics with doxycycline and metronidazole.	All six patients had a favorable early response to to antibiotics, but three suffered with the disease progression, with two of them dying and one of them developing high-grade B-cell lymphoma.	[21]
					Galian stage B: COP with antibiotics, metronidazole and tetracycline, or antibiotics with metronidazole and doxycyclin.		
**38**	F	Turkey	Watery diarrhea, abdominal pain, weight loss, malnutrition, pretibial edema, clubbing and carpopedal spasm.	Hypoalbuminemia, hypokalemia, kypocalcemia, and anemia. Biopsy: lymphoplasmocytic infiltration of the duodenum lamina propria, subtotal vilous atrophy, and chronic gastritis with positive *H*. *pylori* infection. Biopsy after relapse: lymphoma infiltration in deep submucosa of the small intestine.	*H*. *pylori* eradication therapy together with six cycles of CHOP.	Initially CHOP improved clinical condition after the first cycle. After 18 months, symptoms returned and worsened to lymphoma. Final CHOP regimen achieved complete remission and tetracycline maintenance prevented other flare-ups.	[22]
					Repeated six cycles of CHOP. Maintenance therapy with tetracycline (500 mg twice a day) for 56 months.		
**19**	F	Mexico	Diarrhea and abdominal pain for 12 months, nausea, vomiting, fever and night sweats.	Hypogammaglobulinemia, reduced serum IgG and IgM, and low total serum proteins. CT: mesenteric and retroperitoneal lymphadenopathy with diffuse thickening of the small intestinal wall.	Amoxacillin, metronidazole and pantoprazole for 3 weeks, then for 4 months.	Oral antibiotics initially improved clinical condition, but symptoms relapsed 1 month after initial regimen completion. Symptoms.	[23]
**42**	M	Albania	Hematemesis, hypovolemic shock, with marked tachycardia, hypotension, pallor, and diaphoresis.	Low red blood cell count, anemia, leukocytosis, elevated platelets, elevated glucose, lowered lactate dehydrogenase and hypokalemia. Abdominal USG: thickening of the duodenum wall, gallbladder and pancreatic duct distension. Histology post-surgery: high-grade diffuse B-cell non-Hodgkin lymphoma in the duodenum with IPSID signs of mucosal villous abnormalities and diffuse plasmacytic infiltration.	Partial pancreatoduodenectomy (Whipple procedure), followed by six cycles of CHOP and anti-CD20 monoclonal antibody for 5 days every 3 weeks.	Complete remission of iPSID and high-grade B-cell lymphoma after a 9-month follow up.	[24]
**41**	M	Algeria	Diarrhea for 6 months, cachexia, and exudative enteropathy.	Hypoalbuminemia, hypokalemia, metabolic acidosis, elevated serum IgA with reduced IgM and IgG levels, electrophoresis with monomeric α-heavy chain protein precipitation. Positive for *C*. *jejuni* infection. Upper endoscopy: duodenum mucosal edema with white granulations and thickened folds. Capsule endoscopy: shortened and widened villi and crypt hypertrophy in jejunum. Duodenal biopsy: villous atrophy, plasmacytic lamina propria infiltration, positive for α-heavy chain protein.	Doxycycline (200 mg daily) for 6 months.	Symptoms resolved within 1 week; complete remission after a 6-month follow up.	[25]
**15**	M	Australia	Weight loss, anorexia, diarrhea, fatigue and intermittent abdominal pain for 6 months. Steatorrhea, clubbing, and irregular epigastric mass.	Hypoalbuminemia, hypokalemia, elevated lactate dehydrogenase, leukocytosis and elevated platelets, steatorrhea, trichuriais and hepatits B positive. CT: mesenteric lymphadenopathy. Duodenal biopsy: CD45+CD20+ lymphoid cell and plasma cell infiltration of the lamina propria, with the latter extending into the submucosa.	High-dose CHOP, granulocyte stimulating factor, and tetracycline,	Complete remission after a 12-month follow up.	[26]
**36**	M	Australia (indigenous)	Diarrhea for 5 months, weight loss (41 kg) and intermittent abdominal pain, clubbing, and cachexia.	Hypoalbuminemia, hypokalemia, steatorrhea, and elevated serum IgA. Antrum and duodenum biopsies: lymphoplasmacytic infiltration of the lamina propria, chronic inflammation, presence of *H*. *pylori*. Barium meal: thickened nodular folds throughout the jejunum.	Tetracycline (500 mg, 2x/day) for 2 months. One cycle of CHOP.	Tetracycline improved symptoms, but weigh loss and low potassium persisted. After the first cycle of CHOP, patient died of intestinal perforation.	[26]
**57**	M	Japan	Diarrhea and weigh loss for 3 years, pitting edema of the legs, and protein-losing enteropathy.	Hypoalbuminemia, elevated soluble IL-2 receptor, elevated serum IgA, reduced IgM and IgG, and serum protein electrophoresis with polyclonal peaks. Abdominal CT: ascites and edematous stomach wall. Capsule endoscopy: rough mucosa and small intestine villi swelling. Biopsy: lymphocyte and plasma cell mucosal infiltration.	Three cycles of CHOP.	CHOP reduced serum IgA levels and improved symptoms, but patient had heart failure and stopped chemotherapy.	[27]
**34**	M	India	Chronic diarrhea and weight loss for 1 month. History of receiving renal transplant 7 years before and chronic antibody mediated rejection 4 months before. History of cryptosporidiosis.	Anemia, elevated serum creatinine, and positive stool test for giardiasis. Upper gastrointestinal endoscopy: antral gastritis. Duodenal biopsy: chronic inflammatory infiltration of lymphocytes and IgA+ plasma cells in the lamina propria.	For giardiasis, metronidazole and nitazoxanide.	Doxycycline resolved diarrhea, but treatment was insufficient to resolve IPSID.	[28]
					For IPSID, Doxycycline (200 mg daily).		
**20–51, Mean 35.7**	9M, 2F	NR	Diarrhea (n = 10), weight loss (n = 10), abdominal pain (n = 8), finger clubbing (n = 7), anemia (n = 7), and fever (n = 4).	Anemia (n = 1), abnormal urinary D-xylose excretion test (n = 10), increased fecal fat (n = 9), increased serum IgA levels (n = 11), and presence of alpha heavy chain in the serum (n = 11). The total number of patients was 11. Small intestine biopsy: plasma cell infiltration of the lamina propria.	Doxacyclin for 4 weeks, followed by more doxacyclin or tetracycline for 6–12 months. Six cycles of CHOP was given for resistant cases.	Most patients recovered completely. Two developed intestinal obstruction, but remained disease-free after CHOP. Three patients did not respond to CHOP and died.	[8]

Abbreviations: M: Male; F: female; NR: not reported; CT: Computed tomography; PET-CT: Positron emission tomography-computerized tomography; USG: ultrasonography; CHOP: cyclophosphamide, doxorubicine, vincristine, prednisolone, R2-CHOP: rituximab, lenalidomide, cyclophosphamide, doxorubicin, vincristine, and prednisone; COP: cyclophosphamide, vincristine, prednisolone; THP-COP: pirarubicin, cyclophosphamide, vincristine, and prednisolone.

### Clinical features and diagnosis

The most common sites affected by IPSID is the duodenum (63%), followed by the jejunum (17%) [6] and the most common symptoms observed are a chronic diarrhea (53/76, 69.7%) associated or not with weight loss (49/76, 64.4%), nutrient malabsorption (38/76, 50%), abdominal pain (32/76, 42.1%), and digital clubbing (24/76, 31.6%) (Table 2).

**Table 2:** Frequency reported for IPSID clinical features.

Clinical presentation	Number of patients (N = 76)	Reference
Chronic watery diarrhea	53 (69.7%)	[1, 2, 5, 7, 8, 10, 14, 19–22, 25–28]
Weight loss	49 (64.4%)	[1, 2, 5, 8, 13, 15, 16, 18, 21, 22, 26–28]
Malabsorption	38 (50%)	[1, 2, 8, 10, 14]
Abdominal pain	32 (42.1%)	[2, 8, 14, 15, 18, 20, 21, 23, 26]
Finger clubbing	24 (31.6%)	[1, 8, 21, 22, 26]
Vomiting	15 (19.7%)	[2, 16, 23, 24]
Fever	14 (18.4%)	[8, 16, 18, 20, 21, 23]
Anemia	13 (17.1%)	[5, 8, 15, 16, 18, 24, 29]
Decreased appetite	6 (7.9%)	[21]
Edema	5 (6.6%)	[5, 8, 15, 16, 18, 24, 29]
Intestinal perforation	4 (5.3%)	[1, 30]
Steatorrhea	4 (5.3%)	[10, 15, 26]
Cachexia	4 (5.3%)	[1, 7, 25, 26]
Weakness	2 (2.6%)	[7, 26]
Malnutrition	2 (2.6%)	[15, 22]
Intestinal obstruction	2 (2.6%)	[1, 10]
Amenorrhea	1 (1.3%)	[1]
Dehydration	1 (1.3%)	[15]
Carpopedal spasm	1 (1.3%)	[22]
Night sweats	1 (1.3%)	[23]
Anorexia	1 (1.3%)	[26]

**Fig 2:**
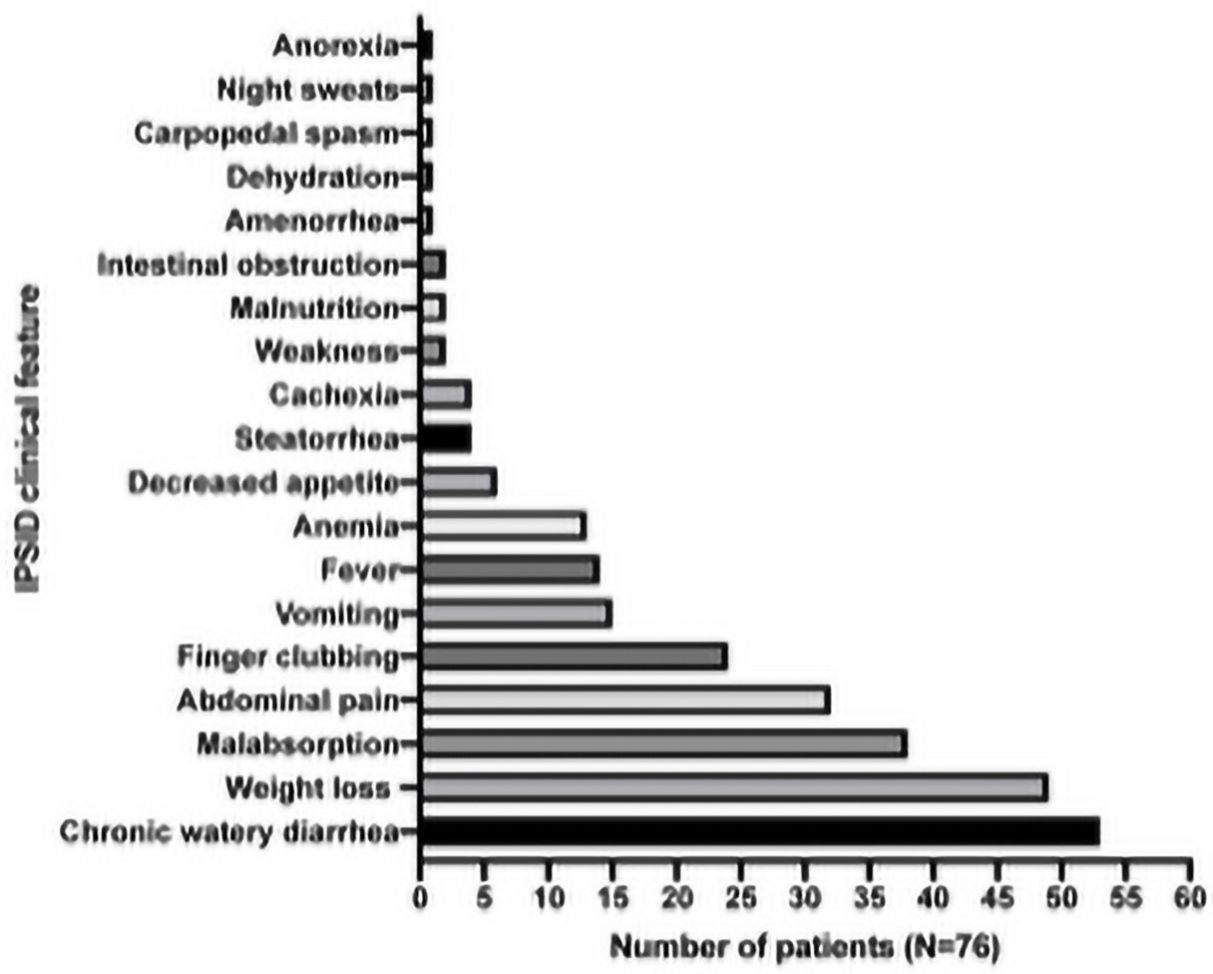
Frequency of the main clinical features reported.

Symptoms may be interspaced in early disease stages, but increase in frequency as it progresses. The time between symptom onset and diagnosis is highly variable and is determined disease stage and progression. Fever is present in late stages, usually low and intermittent. Steatorrhea, cachexia, and weakness are less common, while amenorrhea, dehydration, carpopedal spasm and night sweats are rare. Intestinal obstruction is not a regular feature, but its presence has a bad prognosis as it indicates disease progression into lymphoma [16]. Interestingly, some reports mention a correlation between transplanted kidney patients displaying duodenal villous atrophy and developing IPSID, with persistent diarrhea in 2.16 ± 0.8 years [31]. A visual representation of the most frequent symptoms was reported in this review (Fig 2).

#### Differential diagnose

Mal-absorption syndromes are common and are usually associated with celiac disease and chronic intestinal infection. Patients with IPSID often have a history of being misdiagnosed with celiac disease or chronic intestinal infection because of shared symptoms, being with gluten-free diet with only slight symptom improvement [5]. Celiac disease initial screening consists of measuring transglutaminase and antiendomysial antibodies [11]. It may present mild plasma cell infiltration in the proximal intestine, but IPSID’s mucosal infiltration is more intense and may be present throughout the small intestine [5]. Chronic infectious diarrhea can be ruled out if no pathogenic bacteria are found in the stool culture test, and the presence of certain pathogens like *Campylobacter jejuni* should prompt IPSID investigation if other diseases are unlikely [19].

Lamina propria infiltration with round cells and villus changes are common findings in other diseases and need to be differentiated from tropical sprue, AIDS enteropathy and Whipple’s disease. In these cases, the symptoms added to the histological features are sufficient to perform the differential diagnosis. In tropical sprue, the enterocyte layer often maintains a columnar shape and both enterocyte layer and lamina propria are infiltrated with lymphocytes. In AIDS, the enterocyte layer often maintains a columnar shape and the lamina propria has a dense infiltration of round cells. In Whipple’s disease, enterocytes columnar shape is maintained, keeping a normal luminal surface, while the lamina propria is usually packed with histiocytes detected by positive periodic acid-Schiff staining and has villus widening. However, in IPSID, the enterocytes layer maintains a columnar shape and there is mild lymphocytes and plasma cells, while the lamia propria is packed with lymphocytes that can infiltrate up the muscular layer and frequently causes villous widening [10].

#### Diagnose

With an initial clinical case of chronic watery diarrhea, malabsorption and weight loss, it is important to start with general diagnostic exams to narrow down the etiology for a malabsorption syndrome, and then investigate more specific diseases that are part of the differential diagnosis (e.g. lymphoma, celiac disease, giardia). Laboratory exams to order include: Hemogram, electrolytes, liver enzymes (AST, ALT, gamma glutamyl transferase, alkaline phosphatase), albumin, creatinine, calcium, stool and parasite exam, stool culture test with microbiological identification, *Helicobacter pylori* breath test and serology for IgA, human immunodeficiency virus (HIV), and autoimmune antibodies [5]. If increased levels of IgA are found (>3820 mg/L), bone marrow aspiration should be performed to exclude lymphoma, plasmoma, multiple myeloma and other abnormalities [5]. Percentage of mature and immature plasma cells and morphology should be analyzed in bone marrow smear.

An upper gastrointestinal endoscopy is indicated to investigate the diarrhea and malabsorption. During the exam atrophic nodular mucosa, thickened mucosal folds and edema may be observed. Duodenum biopsies should be obtained for pathological examination [6, 32]. Biopsy histological evaluation is strong diagnostic tool as it shows lymphoplasmocytic infiltration of the lamina propria and villi blunting. IPSID is associated with some radiologic patterns such as enlargement of mesenteric lymph nodes, thickening of the small intestinal wall, dilations and strictures of small bowel loops that may be visualized with a barium contrasted abdominal X-ray or computed tomography [32, 33]. Abdominal ultrasonography can also identify enlarged mesenteric lymph nodes and thick small intestinal wall. Capsule endoscopy may show mucosal edema and villi swelling in small intestine. Double balloon enteroscopy (DBE) is a recently developed endoscopic modality hat can also be used to diagnose small bowel disorders, including IPSID [34].

In the cases analyzed, the most common features presented by IPSID patients were elevated serum IgA (39.5%) with α-heavy chain proteins (35.5%), low or normal serum IgM and IgG (17.1%), anemia (11.8%), lymphocytosis (6.8%), electrolyte disorder with hypokalemia and (9.2%) hyponatremia (1.3%), hypocalcemia (2.6%), and hypoalbuminemia (9.2%) (Table 3). Stool test confirmed steatorrhea (14.5%) and microbiology testing showed a number of pathogens like *Helicobacter pylori* (6.8%) and *Campylobacter jejuni* (3.9%). Computed tomography and upper gastrointestinal endoscopy were the most cited to show alterations in the intestinal mucosa, thickening of the intestinal wall and enlargement of mesenteric lymph nodes.

**Table 3:** Most common exam findings found in IPSID patients.

Exams	Finding	Number of patients (N = 76)	Reference
**Small intestine biopsy**	Lymphoplasmocytic infiltration of the lamina propria	45 (59.2%)	[1, 5, 7, 8, 10, 15– 22, 24– 28]
	Villi blunting / atrophy/ widening	29 (39.1%)	[1, 5, 7, 10, 14–16, 21, 22, 24–26]
**Serum IgA**	Elevated	30 (39.5%)	[1, 2, 8, 15, 19, 25–27]
**α-heavy chain protein**	Present	27 (35.5%)	[1, 7, 8, 19, 25]
**Serum IgG**	Within range or below	13 (17.1%)	[1, 19, 23, 25, 27]
**Serum IgM**	Within range or below	13 (17.1%)	[1, 19, 23, 25, 27]
**Computed tomography**	Mesenteric lymphadenopathy	12 (15.8%)	[7, 15, 18, 20, 21, 23, 26]
	Thickening of the small intestinal wall	11 (14.5%)	[5, 7, 18, 20, 21, 23]
	Hepatomegaly	1 (1.3%)	[7]
**Stool test**	Steatorrhea	11 (14.5%)	[8, 10, 15, 26]
	Occult bleeding	1 (1.3%)	[16]
	Giardiasis	1 (1.3%)	[28]
**Hemogram**	Anemia	9 (11.8%)	[5, 7, 8, 13, 15, 16, 22, 24, 28]
	Leukocytosis (lymphocytosis)	5 (6.8%)	[14, 16, 19, 24, 26]
	Elevated platelets	2 (2.6%)	[24, 26]
**Electrolytes**	Hypokalemia	7 (9.2%)	[7, 15, 22, 24–26]
	Hyponatremia	1 (1.3%)	[7]
**Albumin**	Hypoalbuminemia	7 (9.2%)	[15, 20, 22, 25–27]
**Microbiology testing**	*Helicobacter pylori*	5 (6.8%)	[7, 17, 18, 22, 26]
	*Campylobacter jejuni*	3 (3.9%)	[7, 19, 20]
	*Vibrio fluvialis*	1 (1.3%)	[7]
	*Escherichia coli*	1 (1.3%)	[7]
	*Staphylococcus albus*	1 (1.3%)	[16]
**Calcium**	Hypocalcemia	2 (2.6%)	[15, 22]
**Upper gastrointestinal endoscopy**	Atrophic nodular mucosa	3 (3.9%)	[7, 18, 20]
	Thickening of intestinal folds	2 (2.6%)	[18, 25]
	Edema	2 (2.6%)	[17, 25]
**Barium X-ray**	Intestinal mucosal fold thickening	3 (3.9%)	[15, 18, 26]
	Small intestine dilation	2 (2.6%)	[10, 15]
	Intestinal fold flattening	1 (1.3%)	[20]
**Capsule endoscopy**	Mucosal edema / villi swelling in small intestine	1 (1.3%)	[27]
**Abdominal X-ray**	Lymphadenopathy	1 (1.3%)	[20]
**Colonoscopy**	Nodular mucosa	1 (1.3%)	[7]
**Abdominal ultrasonography**	Enlarged mesenteric lymph nodes	1 (1.3%)	[15]
	Thickening of small intestinal wall	1 (1.3%)	[24]

### Classification

IPSID was classified by Galian et al. in three stages (A, B, and C), depending upon its histopathological features, namely the type of cellular infiltrate and mesenteric nodal involvement [5].

Stage A is characterized by lymphoplamacytic infiltration of the lamina propria with inconstant and variable villi atrophy in the small intestine mucosa [35]. Moreover, there is also plasmacytic infiltration of mesenteric or other abdominal and retroperitoneal lymph nodes, resulting in limited disorganization of the lymph node histological structure [35].

In stage B, there is atypical lymphoplasmacytic infiltration of the lamina propria and atypical immunoblast-like cells spreading to the submucosa with subtotal or total villi atrophy [35]. Mesenteric and other abdominal lymph nodes also are populated with atypical plasmacytic and immunoblast-ike cells severe alteration of the lymph node architecture [35].

Stage C is the most advanced with the proliferation of histiocytes (Hodgkin sarcoma) in all layers of the intestinal wall. This sarcomatous proliferation extends to the mesenteric and other abdominal lymph nodes altering the whole lymph node architecture [35]. In stage C large masses are formed and transformed into malignant lymphoma.

### Treatment

Treatment of patients with IPSID has evolved through time. In the early 1960s, Bracha et al. had described the outcome of the disease when in antibiotic treatment, insinuating an infectious cause [4]. In the late 1980s, the recommendation was that early stage-disease was prescribed tetracycline and metronidazole [36, 37]. These antibiotics were kept for at least 6 months and if there were no marked improvements or no remission within one year, patients were given anthracycline-based combined chemotherapy, like CHOP (cyclophosphamide, doxorubicin, vincristine and prednisolone). For patients that initially presented at late-stage disease, CHOP was given together with antibiotics [1, 6, 32, 33].

More recently, IPSID treatment protocols have been updated to reflect the Galian stages. For stages B and C, a combination of drugs were used, including COP (cyclophosphamide, vincristine and prednisolone), CHOP, nitrogen mustard, MOPP (procarbazine), BACOP (bleomycin, phosphamide, vincristine, prednisolone), CHVP (cyclophosphamide, doxorubicin, teniposide and prednisone) and ABV (doxorubicin, bleomycin, and vinblastine), although the most commonly used is still CHOP [1, 6]. If abnormal cells express CD20, rituximab can be added to the regimen following guidelines for localized extra-nodal marginal zone lymphomas [7, 17, 33, 37].

In addition, supportive measures had to be implemented as most patients were suffering from malabsorption and chronic diarrhea. Supplementation of essential vitamins, minerals and nutrients by parenteral nutrition is key to patient recovery in all chemotherapy regimens.

When the tumor has already developed into a malignant sarcoma with a bulky mass, surgery and radiotherapy present themselves as a palliative strategy to be used before chemotherapy, but not ways to achieve complete remission [6, 33].

Alternatives such as autologous stem cell transplant after high dose chemotherapy and life-long immunosuppression have been proposed, but without consistent data in th records [6, 32]. A comprehensive review of all treatments and their respective outcomes can be consulted in Table 4.

**Table 4:** IPSID treatment and related outcomes.

Galian stage	Category	Treatment	Dose / Duration	Outcome	n	Ref
A	Antibiotics	Tetracycline	-	PR or CR (n = 24), transformation into diffuse large B-cell lymphoma (n = 3)	27	[2]
			-	PR in 1-year follow up. Transformation into malignant lymphoma and death.	1	[15]
			6 months	-	1	[17]
			1 g daily	71% CR, 43.5% 5 year disease free survival	7	[32]
			2 g daily for 6–24 months	33–70% CR	-	[39]
		Tetracycline or metronidazole and ampicillin	-	Overall remission rate of 90% in 2 years, 67% in 3 years	6	[32]
		Tetracycline, ampicillin and/or metronidazole	-	PR, 33–71% of response	-	[6]
			6 months	-	-	[33, 37]
		Tetracycline, ampicillin, metronidazole	42–55 months	PR	-	[36]
		Doxycycline	6 months	CR in 7-year follow up	1	[18]
			200 mg daily for 6 months	CR in 6-month follow up	1	[25]
			200 mg daily	PR	1	[28]
		Amoxacillin, metronidazole and pantoprazole	4 months	PR	1	[23]
		Azithromycin, metronidazole	6 months	CR in 6-month follow up	1	[14]
		*H*. *pylori* eradication therapy: Amoxicillin, clarithromycin, lansoprazole. Followed by amoxicillin, tinidazole, omeprazole and bismuth subcitrate.	500mg, 500 mg, and 30 mg for 7 days. Then, 1 g, NS, NS, and 20 mg for 14 days	CR in 6-month follow up	1	[18]
		*H*. *pylori* eradication therapy	-	-	1	[22]
		*C*. *jejuni* eradication therapy: Amoxicillin, metronidazole, clarithromycin and omeprazole.	1 g twice daily, 500 mg twice daily, 500 mg twice daily, and 20 mg twice daily for 5 months.	CR in 5-month follow up	1	[19]
	Corticosteroid	Prednisone	40 mg daily for 1 month	CR	1	[5]
			30 years	PR for 30 years, death due to total intestinal obstruction	1	[10]
	Supplements	Vitamin D, calcium, magnesium, potassium, iron, folic acid, mecobalamin, and microflora probiotics.	1 month	PR	1	[5]
		Nutritional support, vitamins and mineral supplementation.	6 months	CR in 6-month follow up	1	[14]
		Gluten-free diet	-	PR in 1-year follow up. Transformation into malignant lymphoma and death.	1	[15]
B or C	Surgery	Hemicolectomy and lymphadenectomy	-	CR, 5-year follow up	1	[13]
		Laparotomy	-	Post-surgery complications and death	1	[16]
		Whipple procedure, CHOP and anti-CD20 antibody	5 days every 3 weeks	CR in 9-month follow up	1	[24]
	Chemotherapy	Tetracycline, CHOP	- 500 mg twice daily for 2 months, one cycle	Death by intestinal perforation	1	[26]
		Doxycycline, CHOP	- 10–16 months, 6 cycles	CR (n = 6), intestinal obstruction complication (n = 2), death (n = 3).	11	[8]
		CHOP, CHOP with tetracycline, antibiotics with doxycycline and metronidazole	-	CR (n = 3), transformation into high-grade B-cell lymphoma (n = 1) and death (n = 2).	6	[21]
		COPP, tetracycline	1 g daily for 6 months	CR in 11 patients (68.75%)	16	[11]
		THP-COP, R-P-IMVP16/CBDCA, R-CdA	6 cycles, 2 cycles, NS	PR, transformation into T-cell lymphoma in 2-year follow up and death.	1	[20]
		COP with antibiotics, or metronidazole with tetracycline, or antibiotics with metronidazole and doxycycline	-	CR (n = 3), transformation into high-grade B-cell lymphoma (n = 1) and death (n = 2).	6	[21]
		CHOP	6 cycles	-	3	[2]
			3 cycles	PR, chemotherapy complications	1	[27]
		CHOP, tetracycline, and granulocyte stimulating factor	-	CR in 12-month follow up	1	[26]
		CHOP, maintenance therapy with tetracycline	6 cycles, 500 mg twice daily for 56 months	PR, transformation into lymphoma, CR in 56-month follow up.	1	[22]
		Anthracycline-based chemotherapy	-	Overall remission rate of 90% in 2 years, 67% in 3 years	15	[32]
		CHOP, ABV or CHVP	-	67% 5-year survival	-	[6]
		CHOP, EPOCH, CVAD OR RD-CODOX-M/IVAC	-	Fatal within 2 years	-	[30]
C	Immunotherapy	Rituximab, R2-CHOP	4 cycles, 6 cycles	PR, transformation into diffuse large B-cell lymphoma	1	[7]
		Rituximab	375 mg/m^2^ weekly for 4 weeks	46% CR in patients with lymphoma, 7.7%relapse in 33-month follow up.	27	[40]

Abbreviations: n: number of patients, CR: complete remission, PR: partial remission, NS: not specified; CHOP: cyclophosphamide, doxorubicine, vincristine, prednisolone, R2-CHOP = rituximab, lenalidomide, cyclophosphamide, doxorubicin, vincristine, and prednisone; COP: cyclophosphamide, vincristine, prednisolone; THP-COP: pirarubicin, cyclophosphamide, vincristine, and prednisolone; R-P-IMVP16/CBDCA: rituximab, methylprednisolone, ifosfamide, methotrexate, etoposide, and carboplatin; R-CdA: rituximab and cladribine; EPOCH: etoposide, prednisone, oncovin, cyclophosphamide, hydroxydaunorubicin; CVAD: vincristine, adriamycin, dexamethasone; RD-CODOX-M/IVAC: Rituximab, depocytarabine,cyclophosphamide, oncovin, doxorubicin, methotrexate/ifosfamide, vp16etoposide, aracytarabine.

Table 4 was analyzed according to the following parameters: studies in which the results were provided with sequential follow-up periods, the latest was considered; when the outcome was unavailable or when the number of patients was not reported, the data was not considered for the treatment efficacy percentage calculation.

In stage A, tetracycline alone or combined with ampicillin or metronidazole, was used in 78.2% (43/55) of the patients in this stage. Tetracycline alone achieved 77.1% (27/35) of PR or CR, which was within the 33–71% CR reported in other studies [32, 39]. Doxycycline had 100% PR or CR, but only in 3 patients with only short follow-up periods, which could mean that the patients may have relapsed and were not accounted for. Prednisone also achieved 100% PR or CR, but in only 2 patients, with a 30-year follow-up period in one of them.

In stages B or C, surgery was discarded as a consistent treatment, since it was done in only 3 patients with a different procedure in each one due to other comorbidities. Anthracycline-based chemotherapies were used in 63.5% (40/63) of patients reported in this stage, being the most used therapeutic approach. The overall outcome for patients in anthracycline-based regimens was 62.2% (23/37) PR or CR in their follow-up periods. Other chemotherapy regimens achieved were used to treat 36.5% (23/63) of patients in stages B or C and had 60.9% (14/23) PR or CR rate. Other studies have reported CHOP, ABV or CHVP regimens with overall remission rate of 67% in 5 years [6], which is similar to the values obtained in this review. Antibiotics were associated in 66.7% (42/63) of patients, with a PR or CR rate of 59.5% (25/42). Rituximab was used in 93,1% (27/29) of the cases, specially those with more serious conditions including blown-up lymphomas. Rituximab had a substantial remission rate of 46%, being a very considerable choice among the therapeutic options for stage C.

In sum, stage A has a good clinical response to antibiotics and/or corticoids. Stage B, although treated as stage A in some cases, it can commonly progress to stage C when chemotherapy is not used. Therefore, in order to prevent this unwanted outcome, chemotherapy combined with antibiotics should be offered to treat stage B and C. Moreover, the treatment of stage C with advanced lymphoma should include rituximab.

### Prognosis

When detected in the early stage and appropriately treated, IPSID may be resolved, but relapses are frequently observed [6]. Although the presence of microbial gastrointestinal infection is not a requirement for the establishment of IPSID, pathogens such as *C*. *jejuni* have been associated with IPSID transformation into aggressive diffuse large B-cell lymphoma (DLBCL) in previously healthy individuals [7]. In spite of several treatment regimens, late stages of IPSID (Galian stage C) have a poor prognosis with high mortality rates with a 5-year survival rate of around 67% [1, 6, 38].

The prognostic evaluation of the IPSID can be made with flow cytometry studies. Resistance to chemotherapy can be associated with high S phase of cell cycle and aneuploid DNA indices [39]. On the other hand, a normal S phase and DNA ploidy can be associated with a better response to chemotherapy and, in this case, patients can survive up to 10 years [39].

IPSID patients tend to progress to high grade lymphoma within a few years from the initial symptoms, but Lin et al. reported a case of a 30-year IPSID history without lymphoma conversion, questioning the idea of IPSID as a pre-lymphomatous condition [10]. The explanation for this disease not evolving in this case is still not known and probably involves a different pathogenic mechanism [6].

## Discussion

IPSID is a rare lymphoproliferative disease that cause chronic diarrhea and malabsorption symptoms and evolve into a more severe enteropathy, and even B-cell lymphoma of MALT. The primary goal of this review was to investigate what were the best IPSID treatments in the last 20 years around the world. This goal was significantly constrained by the limited number of literature available on the topic, as IPSID is a rare and neglected disease often associated with developing countries in the Middle East and Africa [23, 32]. This bias was challenged in this review, because although Middle East was the first most reported place of origin for IPSID patients, Europe was the second. Other epidemiological data, like higher male to female ratio [2], and age mean close to the third decade of life was in agreement with earlier studies [2, 21, 32].

The absence of clinical trials specifically for IPSID is another major limitation. Although the Galian stage treatment suggestions made in this review are based on past case reports, research articles and review articles, there was no clinical trial information to corroborate this recommendation. Ideally in the future, medical researchers could collaborate to conduct a controlled clinical study to test different IPSID treatments at each stage using a multicentric approach since local patient recruitment would be a complicated task due the reduced number of patients.

IPSID has confusing diagnostic criteria with many differential diagnosis and complicated treatment regimens, requiring months of antibiotics and/or chemotherapy. This lack of clarity reinforces the need of reviews like this that propose treatment recommendations based on empirical results until structured controlled clinical studies are organized to prove what treatments work best.

Part of the reason why IPSID diagnosis is not straightforward is because IPSID pathogenesis is not fully elucidated. Although it has been associated with chronic antigenic stimulation by gastrointestinal infections, most notably *Campylobacter jejuni* [7], the exact mechanism whereby *C*. *jejuni* precipitates IPSID is still unknown. The main hypothesis is that the exposure to this bacterial toxin in conjunction with previous contact with cholera toxin, can lead to monoclonal lymphoplasmacytic expansion [2, 6, 7, 32]. It has been speculated that *Vibrio cholerae* toxin modulates functional changes in immunological cells, leading defects in B-lymphocytes, T-helper, T-suppressor and natural killer cells. These alterations combined could lead to an acquired deficiency in both cellular and humoral immunity. This hypothesis is supported by reports showing IPSID cases in the same areas affected by cholera epidemic [39].

With an impaired immune system, patients may not be able to effectively clear *C*. *jejuni*. Lymphocytes undergo intense proliferation due to the recurrent antigen stimulation and this intense duplication or the action of *C*. *jejuni* toxins themselves can induce genomic damage in immunoglobulin producing cells [6]. However, no definite causative agent has been identified.

A hypothesis of genetic predisposition was brought on after the development of IPSID in relatives living in different socioeconomic conditions and apart from each other [6, 41]. Elevated levels of alkaline phosphatase were found in members of the same family of a patient diagnosed with this disease [39]. Also, a strong relation between IPSID and human leukocyte antigens AW19, A9 and B12, along with the B blood group, has been reported [6, 7, 42], strengthening the hypothesis for a natural predisposition to IPSID.

Moreover, IPSID pathogenesis starts with immunoglobulin α-heavy chain gene mutations. In this gene, the first constant heavy chain (CH1) domain is responsible for associating α-heavy chains with α-light chains or degrading them by proteasomes. In a mutated gene, this domain is deleted along with a variable heavy (VH) chain component that participates in antigen recognition [6, 32]. Thus, these mutated genes form an abnormal α-heavy chain that cannot be destroyed by proteasomes and alters the immunoglobulin function, leading to immunoglobulin α-heavy chain accumulation. It is interesting to highlight that almost always, α1 is the damaged protein chain [6, 32]. Also, the expression of α-light chain genes is reduced, either by the absence of a transcription factor or by a inhibitory stimulus for the α-light chain transcription [2, 43]. Both these alterations may occur due to clonal abnormalities involving p32 heavy chain locus on chromosome 14 and light chain locus in chromosomes 2 and 22 [32]. There are insertions in the α-heavy chain genes too, but they seem to have no effect since the amino acid sequence encoded by them is not present in the final protein, probably because of an intracellular cleavage [32]. Combined, these factors result in a secretion of truncated α-heavy chain proteins by neoplastic plasma cells, which would not occur without the light chain in normal immunoglobulins and are present even in early stages of the disease [32].

In B lymphocytes, the B-cell receptor has heavy chains in its composition. In IPSID, the truncated α-heavy chain stimulates these receptors, leading to proliferation without an antigen, expanding the number of tumorous cells [6, 30]. Even though mutated B cells have all these abnormalities, their membranes do not present any variable region, allowing them to evade the immune system and contributing to their neoplastic fate [6, 32]. The involvement of Pax5, AML1 and other oncogenes mutations associated with these features previously presented may lead to the full-blown lymphoma due to IPSID, but their role is still uncertain in the pathogenesis [6, 32].

The hallmark lesion of the disease is achieved when intense plasma cell and lymphocyte proliferation and abnormal immunoglobulin secretion associated with an inflammatory process cause structural alterations in the small intestine, such as edema, flattening of the mucosa and villous blunting [2, 6]. Because of the unproven relationship between bacterial infections and IPSID development, antibiotics such as tetracycline, doxycycline and ampicillin have been the most used to treat early IPSID stages, preventing the symptom progression with many cases achieving complete remission. However, since there are other factor contributing to IPSID pathogenesis, it is frequent that patients experience partial remission with symptoms relapse and in some cases, progression into more advanced stages with diffuse large B-cell lymphoma [2, 16]. In more advanced stages, it is recommended the use of anthracycline-based chemotherapy combined or not with antibiotics, and in more extreme cases immunotheraphy can also be used with anti-CD20 monoclonal antibody, rituximabe. Overall, late stags of IPSID do not hold a good prognosis with a patient history marked by several chemotherapy cycles and the need for nutritional support.

## Conclusion

No definite treatment regimen has been stablished for managing IPSID. Therefore, this review characterized IPSID in terms of its clinical features, therapeutic options and treatment outcomes by analyzing English-language articles published in the last 20 years around the world and available in Pubmed. Although IPSID is often cited as a rare condition [6] with diagnostic importance in developing countries, this review identified 76 patients from many continents including Europe, Asia and Oceania, with wealthy countries such as Japan, Italy and Australia included. This information helps to question previously defined paradigms that IPSID is a disease from developing countries. Perhaps, IPSID has been underdiagnosed due to its difficult diagnose and peculiar uncertain association with intestinal bacterial. It would not be hard to imagine that some IPSID cases might have been treated as a bacterial infection and as being treated with antibiotics, has been resolved. Cases that do not curse with bacterial infections and remain untreated can evolve to B-cell lymphoma and thus it is important to raise awareness about this disease and develop an appropriate treatment regimen.

Clinical features from 76 patients were analyzed and classified according to their reported frequencies. To our knowledge, no other review publication has recently analyzed IPSID clinical features focusing on treatment options and outcomes. This information is important because 1) it may aid physicians to diagnose and treat IPSID patients, and 2) it may help public health workers to update clinical guidelines concerning this disease.

IPSID symptoms included chronic watery diarrhea as the most frequently reported symptom, followed by weight loss, malabsorption, abdominal pain, finger clubbing, vomiting, fever and anemia. For triage, the suggested diagnostic investigation includes: hemogram, electrolytes, liver enzymes, creatinine, fecal exam, stool culture, and serology for IgA, HIV, autoimmune antibodies, upper gastrointestinal endoscopy and computed tomography. Lymphocytosis and elevated serum IgA with intestinal endoscopic and radiological alterations suggest IPSID. Diagnosis requires biopsy showing immunohistochemical alterations with lymphoplasmocytic infiltration and villous atrophy of the small intestinal lamina propria.

**Fig 3:**
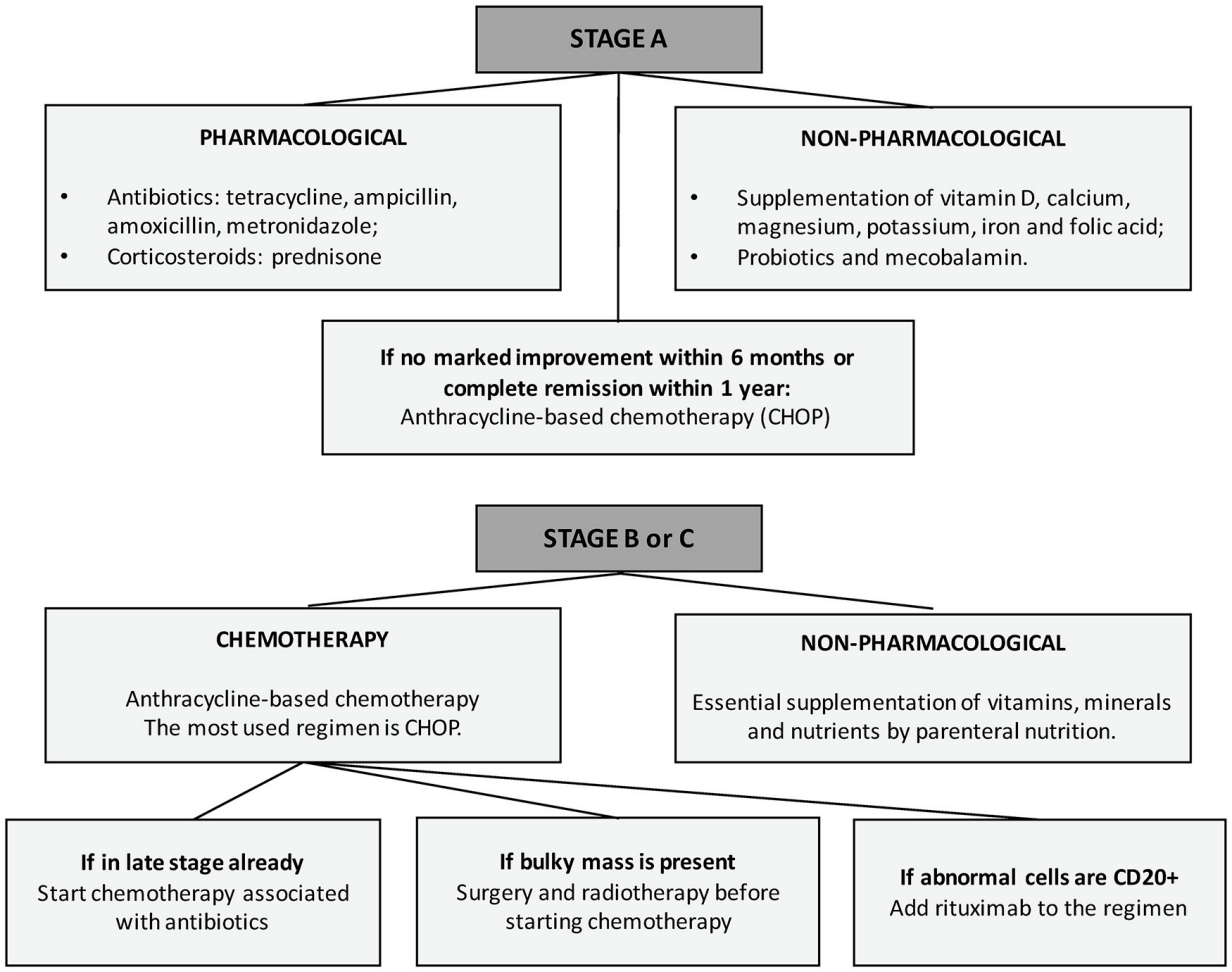
Treatment recommendation flowchart.

A treatment recommendation flowchart was developed based on 1) the most commonly prescribed treatment in all the reported cases, and 2) the best treatment outcomes (Fig 3). In early stages, antibiotics like tetracycline, metronidazole and ampicillin should be used, alone or combined, with a significant therapeutic effect. Due to frequent relapses, a long period antibiotic treatment may be considered. Non-responsive early diseases after 6 months, intermediate and advanced stages require anthracycline-based chemotherapy, which is superior to non-anthracycline-based regimens. Antibiotics previously cited should also be a part in more advanced diseases. The use of rituximab is not well explained and established yet against IPSID, but had satisfactory outcomes. Co-infections with commonly associated pathogens such as *Campylobacter jejuni* must be eradicated if possible.

Early stages of IPSID (Galian stage A) have favorable therapeutic response, so it is important for physicians to consider IPSID as a differential diagnosis in difficult and mysterious cases of chronic diarrhea. Advances stages (B-C) of IPSID confirmed with biopsy have worse prognosis with lower 5-year rate survival, so appropriate therapy is essential.

In this review, it was observed that IPSID seems to be one of the last diagnoses to be considered, which could explain the scarce number of cases often reported in the articles. Another factor to consider is the unequal access to medical services across the globe. Considering Europe was second in the number of cases, perhaps this can be explained by the high quality of medical care in European countries and the overall more egalitarian health system. With an ever more globalized society and a growing number of challenging social conditions related to immigration and refugee camps, physicians from all around the world may be challenged with difficult-to-diagnose chronic diarrheas. In such cases, this review can aid doctors to narrow down their differentials and to diagnose IPSID. Hence, knowledge about this illness will be useful for health professionals and public policy workers everywhere.

## Supporting information

S1 Checklist (PDF)
